# Suicidal risk and executive functions in major depressive disorder: a study protocol

**DOI:** 10.1186/s12888-019-2233-1

**Published:** 2019-08-16

**Authors:** Miquel Roca, Antonio Riera-López del Amo, Pau Riera-Serra, Mª. Angeles Pérez-Ara, Adoración Castro, J. Roman Juan, Mauro García-Toro, Patricia García-Pazo, Margalida Gili

**Affiliations:** 1 Institut Universitari d’Investigació en Ciències de la Salut (IUNICS-IDISBA), University of Balearic Islands, Carretera de Valldemossa, km 7.5, 07122 Palma, Spain; 2 University of the Balearic Islands, Palma, Spain; 3 Department of Nursing and Physiotherapy, University of the Balearic Islands, Palma, Spain

**Keywords:** Major depressive disorder, Suicide, Suicidal attempt, Suicidal ideation, Executive functions, Longitudinal study

## Abstract

**Background:**

Suicide is a serious public health concern. Depression is the main gateway to suicidal behavior. The already established relationship between depression and suicidal risk should now focus on the investigation of more specific factors: recent studies have suggested an association between vulnerability to suicidal behavior and neurocognitive alterations, a nuclear symptom of depression. This project aims to identify alterations in the Executive Functions (EF) of patients suffering a first depressive episode that might constitute a risk factor for suicidal ideation, suicidal attempts and suicide, to allow for more adequate suicide prevention.

**Methods:**

Prospective longitudinal design involving two groups (first depressive episodes with and without alterations in their EF) and four repeated measures (0, 6, 12 and 24 months). The estimated minimum sample size is 216 subjects. The variables and measurement instruments will include socio-demographic variables, clinical variables (age of illness onset, family and personal antecedents, psychopathological and medical comorbidity, suicidal ideation, suicide attempts and completed suicides, severity of depression, including melancholic or atypical, remission of the depressive episode), and neuropsychological variables (EF and decision-making processes evaluated through the Cambridge Neuropsychological Test Automated Battery (CANTAB)).

**Discussion:**

First and foremost, the identification of clinical and neuropsychological risk factors associated with suicidal behavior will open the possibility to prevent such behavior in patients with a first depressive episode in the context of clinical practice. Secondly, interventions aimed at cognitive impairment (in particular: EF) derived from the study may be incorporated into strategies for the prevention of suicidal behavior. Finally, impaired neurocognitive function (even in early stages) could become an identifiable endophenotype or “marker” in clinical and neurobiological studies about suicidal behavior in depressive patients.

## Background

Suicide represents a serious mental health problem. Despite the advances in diagnosis, treatments and better accessibility to health systems suicide rates are increasing worldwide [1]. Current research describes suicidal behavior as the result of an interaction between vulnerability factors and stressful events [2]. Mental disorders are the most notorious vulnerability factors to suicidal behavior, including both attempted and completed suicides amongst adults [3] and young and adolescent population [4]. More specifically, depression is the main risk factor associated with suicide [5, 6]. In spite of depression having been defined primarily as a mood disorder recent evidence shows that cognitive disturbances are a nuclear part of the symptomatology [7–9], being already included in the nosological definitions of the disorder [10]. In recent years an increasing number of papers, from the fields of neuroscience and neuropsychology, have established that between 20 and 60% of depressed patients show different alterations in executive functions (EF) [11]. Trivedi and Greer [12] analyzed 12 papers, systematic reviews and meta-analyzes, that included comparisons between cognitive performance of depressed patients and healthy subjects and concluded that there is enough evidence supporting the fact that cognitive alterations appear in depression, mainly in attention, psychomotor speed, and EF. Another meta-analysis [13], showed that between 20 and 40% of depressed patients manifest poor performance in different neuropsychological measures of EF. These cognitive alterations appeared to be determinant in evolution and prognosis [14], higher relapse rates [15] and functional and occupational capacity [16, 17]. Thus, achieving the remission of cognitive impairment would be essential for a complete functional recovery in depressed patients [18].

Over the past years, cognitive rigidity was closely related to suicidal behaviors [19, 20] until King et al. [21] indicated that this rigidity could be the result of certain alterations in EF, which could be understood through more complex models [22]. EF allow us to control and direct our behavior [23], and due to their role as behavior controller, they are relevant in the appearance of self-injuring behavior, including suicide and suicide attempts, when confronting stressful situations. Inhibition, Working Memory and Cognitive Flexibility are considered to be the cores of EF [24, 25], and from their interaction a higher-order set of EF such as reasoning, problem solving, and planning emerges [26]. Alterations in these EF would represent an obstacle in engaging adaptative coping behaviors, stop ruminating and shifting to more positive thinking, among other behaviors strongly linked to suicidality [27–29]. Decision making [30] and impulsivity [31] have also been found to be related to suicidality.

Practically all the investigations into the relation between cognitive function and suicidality in depressive patients has occurred since the year 2000 [32] through cross-sectional studies, and usually during hospitalization after a suicide attempt. In almost all cases the collection of information is obtained retrospectively by interviews and clinical histories [33, 34]. Only a few of these studies have specifically addressed the relationship between alterations in EF and suicidal ideation [35, 36]. In the first study [35], 28 depressive patients without suicidal ideation were compared with 25 depressive patients with such ideation, with the latter offering significantly lower performances in different EF tests, related to the aforementioned “cognitive rigidity” (Wisconsin Card Sorting Test and Trail Making Test). This suggests dysfunctions in decision making related to the frontal lobe. In another study [36], 14 depressive patients without suicidal ideation and 15 depressive patients with suicidal ideation and a suicide attempt admitted to a psychiatric unit were compared with 29 healthy controls. Only the depressed patients with suicidal ideation showed executive dysfunctions, particularly in the decision-making tasks. In a more recent study by Ming-Chou Ho et al. [37] where they evaluated the EF of 34 MDD attempters, 36 MDD non-attempters, and 55 healthy controls their findings were consistent with previous review and meta-analytic studies. To date there is no longitudinal study following first depressive episodes, which examine whether alterations in one or more EF, or other cognitive alterations, will predict suicidal ideation, attempts or subsequent consummate suicidal behavior. This is the great limitation of studies published to date. Thus, we propose the present longitudinal study to evaluate the alterations in EF found by previous cross-sectional studies directly after a first MDD diagnosis and for the next 24 months through a series of 4 assessments after baseline, 6, 12 and 24 months.

## Methods

### Objectives

To evaluate the alterations in EF found by previous cross-sectional studies directly after a first MDD diagnosis and for the next 24 months through a series of 4 assessments. The main objective is to establish whether there is a relation between suicidal behavior and ideation and alterations in EF. Completed suicides, suicide attempts and suicide ideation will be registered among other variables such as medical history, psychopathological comorbidities with MDD and history of familiar suicide. This study will contribute towards the goal of developing predictive models and enhance suicide prevention efforts.

### Design

Longitudinal study with 4 data collection points at baseline, 6, 12 and 24 months. Participants will be assigned to two different groups with EF alterations and without EF alterations depending on their EF scores as measured by CANTAB.

### Measurements

1. *Sociodemographic questionnaire*. Relevant sociodemographic information regarding gender, date of birth and age, marital status, education level and occupational status.
2. *Clinical history*. Previous and/or concurrent medical conditions will be informed by the health centers.
3. *Mini International Neuropsychiatric Interview (M.I.N.I.)* [38] Comorbid psychopathologies will be evaluated using this diagnostic interview. This interview is divided into modules, each one of them comprising a diagnostic category. The Spanish version will be used [39].
4. *Columbia- Suicide Severity Rating Scale (C-SSRS)* [40] version validated for Spanish population [41]. C-SSRS is an assessment tool that evaluates suicidal ideation and behavior. It provides a Suicidal Ideation Score (ranking 1 to 5), Suicidal Behavior and Self-Injurious Behavior without Suicidal Intent. Suicide or suicide attempts that might take place between follow-up assessments will be reported by the primary care centers.
5. *Inventory of Depressive Symptomatology Self-Rated (IDS-SR30)* [42] version validated for Spanish population [43] is a diagnostic tool consisting of 30 items comprising the 9 nuclear symptoms of depression according to DSM5 criteria. It also evaluates anxiety and irritability symptoms and melancholic and atypical depressions. A cut-off score of 18 represents mild depressive symptomatology. MDD will be considered in remission when IDS-SR30 scores at follow-up assessments (at 6, 12 and 24 months) are equal or inferior to 13.
6. *Cambridge Neuropsychological Test Automated Battery (CANTAB*) [44] will be used to evaluate alterations in EF. Specifically, the following tests will be used: *Motor screening Task* (MOT); Sensorimotor function and comprehension. *Spatial Working Memory* (SWM), which measures working memory and strategy by asking the participants to find a hidden target inside boxes. *One Touch Stockings* (OTS); Planning and working memory measured through latency and accuracy of participant’s responses when asked how many moves are required to match two sets of colored balls. *Stop Signal Task* (SST); Response inhibition, and *Cambridge Gambling Test* (CGT); Decision making. Tests have been validated in behavioral and psychopharmacological studies on healthy human volunteers and in a range of patient groups [9].

### Recruitment and study settings

Participants will be recruited from primary health care centers of the Balearic Islands (Spain), mental health units, and external consultations from the hospitals: Son Espases, Son Llàtzer, Manacor, Inca and Joan March from Majorca; Can Misses from Ibiza; and Mateu Orfila from Minorca. Each one of the these centers will recruit participants that meet all the eligibility criteria. When a potential participant comes in contact with one of the collaborating centers the medical staff will try to persuade them to enter the study, and if they do, then sign the informed consent form. Afterwards, medical staff will fill in a brief reference form, indicating that the participant meets the eligibility criteria and will inform the main PI. With that information the University of the Balearic Islands researchers will contact the participants to schedule a date to conduct the baseline assessment. All evaluations will take place in the participants’ centers. Baseline assessment, after verifying that participants meet eligibility criteria and answering any doubts that the participants may have and confirming that written informed consent is provided, will consist of an interview to obtain socio-demographic information (such as age, gender, educational and socio-economic status) familiar history of suicidal ideation and suicidal behavior. Baseline evaluation will also include the MINI International Neuropsychiatric Interview (MINI 5.0) in order to confirm the diagnosis of depression, whether it is the first one and if there are other comorbid disorders. The participants’ suicidal ideation and/or suicidal behaviors will be assessed using the Columbia–Suicide Severity Rating Scale (C-SSRS). Finally, EF will be evaluated using the CANTAB battery tests. The estimated duration of baseline assessment is 80 min. Participants will be offered a 30 min’ breakout in case they need it. All assessments will take place between 8 am and 3 pm to control mood variability across the daytime. After baseline, three more data collection points will take place at 6, 12 and 24 months, with the content being the same in these assessments as the ones listed in the baseline assessment. The schedule of enrolment and assessments of the study can be seen in Table 1.

**Table 1:** Schedule of enrolment and assessments

	STUDY PERIOD				
Timepoint	T0	T1	T2	T3	T4
	Pre-Enrolment by medical staff	Baseline assessment and enrolment	6 months	12 months	24 months
Eligibility screen	X	X			
Informed consent	X	X			
MINI		X	X	X	X
IDS-30		X	X	X	X
C-SSRS		X	X	X	X
CANTAB test		X	X	X	X

### Eligibility criteria

Inclusion criteria for the study are that participants must be adults aged between 18 and 65 years old, diagnosed with major depressive disorder according to psychiatric DSM5 criteria, and with a score in the Inventory of Depressive Symptomatology- Self-Rated (IDS-SR30) of at least 18. They must also be able to understand and sign the informed consent form. The exclusion criteria are: to have suffered previous episodes of mayor depression, to suffer any kind of medical condition that may cause cognitive impairment, to present any kind of neurologic illness or have suffered from cranioencephalic traumatisms, to present psychotic symptoms or be diagnosed with borderline personality disorder, and so, be treated with antipsychotic or euthymic drugs or have received electroconvulsive therapy at any moment in their lives. They must also be unable to understand and complete the different tests of the study.

### Sample size

Sample size calculations are based on the main result measure, the ideation severity sub-scale of the Columbia Suicide Severity Rating Scale (with value ranking from 1 to 5). A sample size of 180 participants has been established assuming equal variance between groups with different EF, an alpha bilateral error of 5%, an 80% power and an estimated effect size of 0.25 in relation with ideation and suicide attempts. This will allow for the detection of a difference of 0.5 points on the sub-scale (assuming a standard deviation of 2.0). Attrition rate is expected to be a 20%, therefore the sample size is 216 participants. The estimate effect-size of 0.25 is considered low [45] which will allow to find effect-sizes that might be missed using smaller samples.

## Discussion

To our knowledge, this will be the first study that longitudinally compares cognitive functioning in depressed patients after a first MDD episode and the emergence of suicidal ideation. Nowadays, different research lines are aiming to elucidate whether cognitive alterations disappear or not during the phases of remission, imply stable or permanent deficits and, whether they depend on the severity, number of episodes or depressive subtype, among other variables. More evidence is needed to determine whether cognitive alterations are a “state”, a “trait” or a “scar”. If the cognitive alterations were a “state” they would only be present during the acute phases of the disease, normalizing when reaching remission. Systematic reviews do not support the existence of this “state” marker [14, 46]. Cognitive alterations as a “trait” would imply that their existence and persistence would imply greater vulnerability to a first episode or subsequent recurrent episodes. Studies in the general population have suggested that problems in episodic memory would be prior to a first episode [47, 48]. Finally, the “scar” hypothesis implies that the cognitive alterations observed in the first episodes would persist over time, even if residual or clinical remission is reached. Successive recurrent episodes may increase this “scar” and consequent cognitive symptoms [49]. In previous published studies we found that cognitive alterations were still present 6 months after clinical recovery was achieved [8, 50]. Therefore, it is essential to describe the cognitive profile of patients with a first depressive episode and analyze the evolution throughout the disease in order to deliver effective interventions that take care of cognitive symptoms.

Translational research of depressive disorders for the detection of MDD should identify clinical profiles already in the first MDD episodes with symptoms that respond to specific cognitive alterations that research has shown to predict suicidal behavior. In this early detection, the data from basic, genetic or neuroimaging, or neuropsychological research, given its complexity, can only be applied when they are translated into symptoms or clinical patterns detectable in the short time of clinical consultations. Given that around 50% of patients who commit suicide have visited the Primary Care physician 3 months before their self-injurious behavior, 40% in the immediately preceding month and 20% just 1 week before [51] it is vital to develop tools that allow for a quick identification of risk profiles.

### Limitations of the study

Even though the present study overcomes the limitations of previous studies it still has some limitations. All cognitive functions will not be explored; the study will focus on the EF in which some alteration has been detected in depressive patients or been linked to suicidal behavior in depression. Personality will not be evaluated in the study. Even though it is going to be a controlled variable, the pharmacological or psychotherapeutic treatments administered will not be the same for all patients included in the study. There might be a sample loss based on the 24-month follow-up time proposed, which at the same time constitutes the greatest strength of the study. For this reason, sample size has been increased by 20%. Before starting, meetings will be held with the doctors of primary health care centers, mental health units, and external consultations to improve recruitment homogeneity and reduce the biases that have been detected in this type of studies.

## Data Availability

Datasets supporting the conclusions of all articles derived from this research will be available in online specialized repositories, still to be chosen.

## Abbreviations

CANTAB: Cambridge Neuropsychological Test Automated Battery
EF: Executive Functions
MDD: Major Depressive Disorder
MINECO: Spanish Ministry of Economy and Competitiveness
PI: Principal Investigator
